# Emerging roles of pseudogene-derived lncRNAs in cancer stem cells: Non-coding clues and therapeutic targets in cancer medicine

**DOI:** 10.1016/j.gendis.2025.101793

**Published:** 2025-08-07

**Authors:** Seyed Taha Nourbakhsh, Fatemeh Mohamadhashem, Elahe Soltani Fard, Faezeh Mohamadhashem, Abdolreza Daraei

**Affiliations:** a Cellular and Molecular Research Center, Basic Health Sciences Institute, Shahrekord University of Medical Sciences, Shahrekord 8815713471, Iran; b Department of Internal Medicine, Sina Hospital, Tehran University of Medical Sciences, Tehran 1461884513, Iran; c Student Research Committee, Shahrekord University of Medical Sciences, Shahrekord 8815713471, Iran; d Department of Molecular Medicine, School of Advanced Technologies, Shahrekord University of Medical Sciences, Shahrekord 8815713471, Iran; e Department of Medical Genetics, School of Medicine, Tehran University of Medical Sciences, Tehran 8815713471, Iran; f Cellular and Molecular Biology Research Center, Health Research Institute, Babol University of Medical Sciences, Babol 47176-47745, Iran; g Department of Medical Genetics, School of Medicine, Babol University of Medical Sciences, Babol 47176-47745, Iran

**Keywords:** Cancer stem cells (CSCs), ceRNA network, Pseudogene-derived lncRNAs, Signalingpathways, Tumorigenesis

## Abstract

Cancer stem cells (CSCs), progenitor tumor cells with stemness characteristics, play key roles in cancer's onset, progression, metastasis, relapse, and chemotherapy resistance. While the exact molecular mechanisms of CSC development are not fully understood, recent research has revealed regulatory pathways of their generation with the weighty involvement of non-coding RNAs. It has been found that some pseudogenes are transcribed to long non-coding RNAs (lncRNAs), which are functionally and structurally similar to typical lncRNAs with biological functions including sponge miRNAs, antisense RNA, and interactions with proteins. Outstandingly, various *in vitro* and *in vivo* evidence have demonstrated that dysregulation of pseudogene-derived lncRNAs is directly involved in the development of CSCs in different cancers, mainly through functioning as miRNA sponges for modulating CSC-related signaling pathways. Therefore, researchers have suggested that research in this field can reveal hidden aspects of CSC development and can also open a new window for developing novel cancer therapeutic and diagnostic targets. In this review, we comprehensively address the recent findings of previous studies on the dysregulated roles of pseudogene-derived lncRNAs in directing and generating CSCs in various cancers. Also, their clinical capacities in terms of biomarkers, diagnosis, and treatment for cancer will be discussed.

## Introduction

Cancer stem cells (CSCs), also referred to as tumor-initiating cells and cancer-initiating cells, are a subpopulation of tumor cells to which the tumor behaviors, including growth rate, metastasis potency, and resistance to treatment, are attributed.^1^ Dick and colleagues originally discovered CSCs in acute myeloid leukemia with two key characteristics, including self-renewal and differentiation into mature terminal malignant cells.^2^ The role of CSCs in developing and maintaining tumors in different hematological and solid malignancies was evidenced by several studies.^3^ Phenotypically, numerous cell surface or intracellular markers have been used to identify CSCs in hematological and solid tumors, including aldehyde dehydrogenases (ALDHs), transforming growth factor-beta (TGF-β), β-catenin, Nanog, cluster of differentiation 24 (CD24), CD44, CD90, and CD133.^4^, ^5^, ^6^, ^7^ Embryonic stem cells rely on multiple transcription factors associated with pluripotency to regulate organ development and influence cell fate. Examples include octamer-binding transcription factor 4 (OCT4), SRY-related HMG box 2 (SOX2), NANOG, Kruppel-like factor 4 (KLF4), and MYC.^8^ By transiently overexpressing these factors, somatic cells can be reprogrammed into induced pluripotent stem cells.^9^^,^^10^ Growing evidence suggests that pluripotent transcription factors are largely suppressed in adult tissues but are overexpressed in aggressive cancers.^8^^,^^11^ This overexpression plays a role in regulating the biological functions of CSCs and imparts numerous distinctive traits to them. Transcription factors in CSCs, including OCT4, SOX2, and NANOG, are recognized as critical regulators. These factors contribute significantly to the self-renewal, proliferation, and differentiation of CSCs by interacting with specific DNA sequences and modulating the expression of stemness-related genes. This regulation ultimately drives tumor initiation, invasion, and metastasis.^12^, ^13^, ^14^

The origin of CSCs is a matter of controversy; three main theories are proposed in this regard. The first argues that CSCs are derived from normal stem cells after various genetic mutations in response to environmental stimuli. According to the second hypothesis, progenitor cells that are not yet fully differentiated and have undergone mutation during tumorigenesis are the source of CSCs. The third theory proposes that fully differentiated cells carrying cancerous mutations lead to the formation of CSCs. What all three theories share is that tumor-related mutations play a key role in both malignancy and the maintenance of stem-like characteristics of the CSCs.^15^, ^16^, ^17^

In the tumor tissue mass, the microenvironment or niche plays a pivotal role in maintaining the stemness of CSCs, where, apart from intracellular molecular interactions, intercellular communication ensures the function and emergence of CSCs for the growth and development of cancer.^18^ Investigations on the CSC niche have shown that a range of cells, including cancer-associated fibroblasts, endothelial cells, immune cells, and mesenchymal stem cells, are present in tumor-specific microenvironments.^19^ These tumor microenvironmental cells support CSC development and differentiation and promote tumorigenesis, angiogenesis, invasion, and metastasis by releasing growth factors or activating survival pathways through cell–cell contacts.^20^ Furthermore, the microenvironment of CSCs has a significant role in resistance to therapy and relapse.^20^, ^21^, ^22^, ^23^ In normal stem cells, several highly regulated signaling systems are embedded to maintain their biological function and homeostasis; numerous signaling pathways are involved in the survival, proliferation, differentiation, and maintenance of self-renewal capacity of stem cells through the regulation of specific gene expression, which have been revealed to be inappropriately hyperactivated or inhibited in cancer during the development of CSCs.^9^^,^^24^ Among the most prominent of these signaling pathways are the TGF-β, NOTCH, Wnt, Janus kinase (JAK)/signal transducers and activators of transcription (STAT), Hedgehog, phosphoinositide 3-kinase (PI3K)-protein kinase B (AKT)-mammalian target of rapamycin (mTOR), nuclear factor kappa B (NF-kB), and peroxisome proliferator-activated receptor (PPAR).^9^^,^^24^^,^^25^

Recent studies have demonstrated that some expressed pseudogenes are dysregulated in tumorigenesis by producing a class of long non-coding RNAs (lncRNAs) called pseudogene-derived lncRNAs, which play a significant role in hindering the above-mentioned signaling pathways during the formation of CSCs in a variety of malignancies. It should be noted that pseudogenes have long been considered “junk genes” due to the presence of disabling mutations that prevent them from being transcribed or translated.^26^, ^27^, ^28^ Relying on advancements in genome-wide platforms, recent studies claim that many pseudogenes are transcriptionally active, encoding an important type of lncRNA transcript that has a role in some human diseases, including cancer.^29^, ^30^, ^31^, ^32^, ^33^ lncRNAs are non-coding RNAs with a length of more than 200 bases with no code for protein production.^34^^,^^35^ It is well known that lncRNAs have various biological functions at both transcriptional and post-transcriptional levels of gene expression.^36^, ^37^, ^38^ One of the most important roles of lncRNAs is to sponge microRNAs (miRNAs) to inhibit the translation of messenger RNA (mRNA), which in turn stimulates the production of protein from mRNA by ribosomes; in this phenomenon, they are referred to as competing endogenous RNAs (ceRNAs).^39^^,^^40^ Similar to lncRNAs, pseudogene-derived lncRNAs act as ceRNAs to prevent miRNAs from binding to their mRNA targets.^41^ Through this mechanism, dysregulated pseudogene-derived lncRNAs play one of their most important roles in tumorigenesis.

This review aims to provide a comprehensive overview of the most important signaling pathways involved in regulating CSC development through pseudogene-derived lncRNAs, classification and biological functions of pseudogene-derived lncRNAs, and recent discoveries regarding the central roles of dysregulated pseudogene-derived lncRNAs in the formation and development of CSCs in various human tumors. Additionally, we will explore their potential clinical applications as therapeutic, diagnostic, and prognostic targets in oncology.

## Signaling pathways involved in regulating CSC development by pseudogene-derived lncRNAs

Recent studies show that lncRNAs manipulate some signaling pathways in CSCs by inappropriately activating or suppressing them, hence altering their survival, proliferation, self-renewal, and differentiation characteristics; the Wnt, JAK-STAT, TGF-β, extracellular signal-regulated kinase 1/2 (ERK1/2), and PI3K/AKT/mTOR are among the most important signaling pathways involved.

## Wnt signaling pathway

The Wnt signaling pathway is involved in several early developmental processes, including cell polarity, proliferation, differentiation, and cell fate determination.^42^ Well conserved during evolution, with 19 Wnt ligands and more than 15 receptors, it is considered a highly complex signaling pathway.^43^ It is composed of two distinct pathways: the canonical pathway (through the Frizzled (FZD)-low-density lipoprotein receptor-related protein 5/6 (LRP5/6) receptor complex), the Wnt/β-catenin signaling pathway, and the non-canonical pathway, the Wnt/calcium and Wnt/planar cell polarity (PCP) signaling pathways.^44^^,^^45^ In canonical Wnt signaling, β-catenin is phosphorylated by glycogen synthase kinase 3 (GSK3) in the absence of Wnt ligands. As a result, beta-catenin is degraded by ubiquitination, preventing its translocation from the cytoplasm to the nucleus.^46^ In the presence of Wnt signaling, the binding of LRP5/6 and FZD suppresses the function of the Axin complex and the phosphorylation of β-catenin, allowing β-catenin to enter the nucleus and subsequently connect to LEF/TCF to create a complex that then recruits cofactors to start the transcription of downstream genes.^47^ The aberrant Wnt/β-catenin signaling plays a role in the stemness of CSCs and promotes CSC properties such as expression of cell surface markers, self-renewal, and tumorigenicity.^48^ Furthermore, resistance to therapy mediated by CSCs is regulated by the Wnt/β-catenin pathway. For example, sorafenib resistance in hepatocellular carcinoma is caused by protein tyrosine kinase 2 (*PTK2*) promoter hypomethylation, which also causes *PTK2* overexpression and stimulates the signaling pathway of Wnt.^49^ Conversely, several Wnt factors trigger non-canonical Wnt signaling pathways, referred to as the PCP or Wnt/calcium pathway, which control asymmetrical cell division, cell movement, and cell polarity.^50^, ^51^, ^52^ Despite being less researched than the canonical pathway, noncanonical Wnt signaling could potentially contribute to the development of tumors through the noncanonical Wnt ligand Wnt5a. The Wnt5a signaling pathway plays an essential role in controlling fundamental developmental processes, such as self-renewal, cell division, differentiation, cell migration, and adhesion.^45^^,^^53^ According to recent research, Wnt5a signaling has a role in controlling the self-renewal of CSCs as well as the division, invasion, and migration of cancer cells.^45^^,^^53^

## JAK-STAT signaling pathway

The JAK/STAT signal transduction is a crucial pathway for many growth factors and cytokines, and therefore for many biological processes, including cell division, proliferation, apoptosis, and immune control.^54^^,^^55^ Tyrosine kinase JAK, transcription factor STAT, and receptors (which bind to chemical signals) are the three essential components of the JAK/STAT system. The JAK family has four members: JAK1, JAK2, JAK3, and TYK2.^56^^,^^57^ Activated JAK tyrosine kinases send out regulatory signals when cytokines attach to their receptors.^58^ So far, seven STAT family members have been identified: STAT1, STAT2, STAT3, STAT4, STAT5a, STAT5b, and STAT6.^57^ Several cytokines and related JAKs can activate any member of the STAT family,^59^ by triggering a variety of ligands, generally cytokines like interferons and interleukins. In the first step, the receptor-associated JAKs are brought into proximity to each other by the dimerization of the receptors. Following that, the JAKs phosphorylate one another on tyrosine residues, making the kinase domains more active. The tyrosine residues on the receptor are then phosphorylated by the activated JAKs to create a binding site for STATs. When STATs bind to the phosphorylated tyrosines on the receptor, JAKs phosphorylate STATs, and then STATs dissociate from the receptor and form homodimers or heterodimers. Finally, these dimers get into the nucleus as active transcription factors, where they interact with DNA-binding sites and control gene transcription.^60^, ^61^, ^62^, ^63^ According to studies, CSCs from a variety of malignancies, including the prostate, glioma, breast, and blood, display aberrant activation of the JAK/STAT pathway.^64^, ^65^, ^66^, ^67^ Furthermore, it has been shown that this pathway plays a role in the metastasis, self-renewal, and tumorigenesis of CSCs.^9^ This is why the inhibitors of this pathway are now a well-known class of anti-neoplastic agents.^68^

## TGF-β signaling pathway

The TGF-β signaling pathway is involved in several cellular functions from the early stages of development to adulthood, including cell proliferation, cell differentiation, apoptosis, and cellular homeostasis.^69^ It has been discovered that the genome of mammals encodes 33 polypeptides corresponding to the TGF-β family.^70^^,^^71^ Members of the TGF-β family include activins, bone morphogenetic proteins (BMPs), growth differentiation factors (GDFs), Nodal, and TGF-β.^72^ TGF-β superfamily ligands bind to the cell membrane's type I (TβRI) and type II (TβRII) serine and threonine kinase receptors, initiating the TGF-β signaling cascade.^73^ TβRII phosphorylates TβRI, and the TβRII-TβRI receptor complex subsequently phosphorylates receptor-regulated Smads (R-Smads) to activate specific genes in the nucleus.^74^ Recent investigations have shown that TGF-family signaling plays pivotal roles in CSC maintenance and development in different cancers.^69^^,^^75^^,^^76^ Overactivation of the TGF-β signal pathway is said to increase the stemness of CSCs in triple-negative breast cancer; therefore, it is proposed that the prognosis of triple-negative breast cancer can be improved by combining traditional chemotherapy drugs with TGF-β inhibitors.^77^ Furthermore, TGF-β is also reported to regulate stemness, self-renewal, and resistance to chemotherapy in liver cancer.^78^

## PI3K/AKT/mTOR signaling pathway

The PI3K/Akt/mTOR signaling pathway plays a vital role in essential cellular processes such as proliferation, transcription, translation, cell survival, and angiogenesis.^79^^,^^80^ The PI3K heterodimer, which is a group of lipid kinases, is a key component of this pathway.^81^ Due to the presence of the p38 regulatory and p110 catalytic subunits, PI3K exhibits serine/threonine kinase and phosphatidyl-inositol kinase activities.^82^ Also known as protein kinase B (PKB), serine/threonine kinase Akt is an important PI3K downstream molecule activated in response to PI3K. AKT1, AKT2, and AKT3 are the three expressed isoforms of AKT.^83^^,^^84^ The mTOR complex, a conserved serine/threonine kinase, is one of the important downstream target genes of AKT. The mTOR divides into two structurally and functionally unique complexes, mTORC1 and mTORC2.^85^ In comparison to other principal signaling networks, abnormalities in the PI3K/AKT/mTOR pathway are observed more in human malignancies.^86^, ^87^, ^88^, ^89^ Additionally, emerging data indicate a link between the CSC metabolism and the mTOR signaling system.^90^^,^^91^ The pathway of mTOR has a role in both epithelial-to-mesenchymal transition and the proliferation of ovarian cancer cells.^92^ Furthermore, the migration and invasion of pancreatic and prostate CSCs are enhanced by activation of this signaling pathway.^93^^,^^94^

## MAPK/ERK signaling pathway

The mitogen-activated protein kinase (MAPK) pathway is one of the key signaling pathways involved in converting extracellular signals into cellular responses. ERK1/2, c-Jun NH2-terminal kinase, and p38 kinase are members of the mammalian MAPK family.^95^^,^^96^ ERK1 and ERK2 are serine–threonine kinase proteins that contribute to the regulation of essential cellular functions, including proliferation, differentiation, migration, survival, and apoptosis, through their involvement in the Ras-Raf-MEK-ERK signaling cascade.^97^ The ERK1/2 signaling pathway is typically activated upon the binding of extracellular ligands, such as growth factors, to a receptor tyrosine kinase (RTK) located in the plasma membrane. After the binding of a ligand, the receptor dimerizes and then undergoes autophosphorylation of its intracellular tyrosine residues. The phosphorylated residues serve as binding sites for the adaptor protein growth factor receptor-bound protein 2 (Grb2), which subsequently binds to the SOS (guanine nucleotide exchange factor). Subsequently, SOS changes Ras-GDP into Ras-GTP, which is now active. The activated Ras then attaches to Raf and triggers its activation. Finally, mitogen-activated extracellular signal-regulated kinase 1/2 (MEK1/2), which is phosphorylated by Raf, activates ERK1/2.^98^, ^99^, ^100^, ^101^ The outcome is the phosphorylation of numerous crucial targets by ERK1/2.^102^^,^^103^ By regulating the development of CSCs, the ERK1/2 pathway contributes significantly to the development of several cancers.^104^, ^105^, ^106^ For example, Ding et al demonstrated that lncRNA H19 inhibition might contribute to the induction of oxidative stress and the reduction of chemoresistance of CD133^+^ CSCs in hepatocellular carcinoma by blocking the MAPK/ERK signaling pathway.^107^

## Biogenesis and classification of lncRNAs

**Figure 1:**
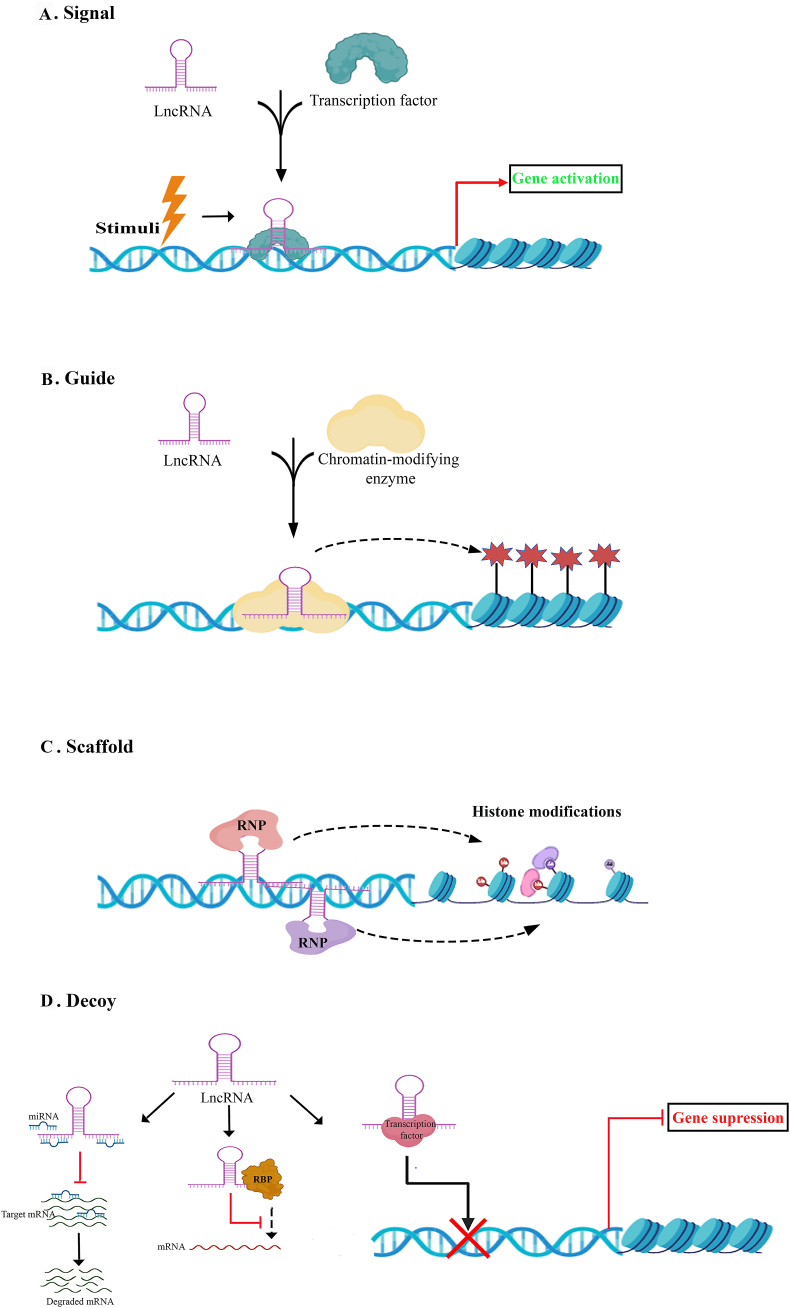
Functional mechanisms of lncRNAs. lncRNAs regulate gene expression through four distinct functional pathways. **(A)** Signal. **(B)** Guide. **(C)** Scaffold. **(D)** Decoy.

**Figure 2:**
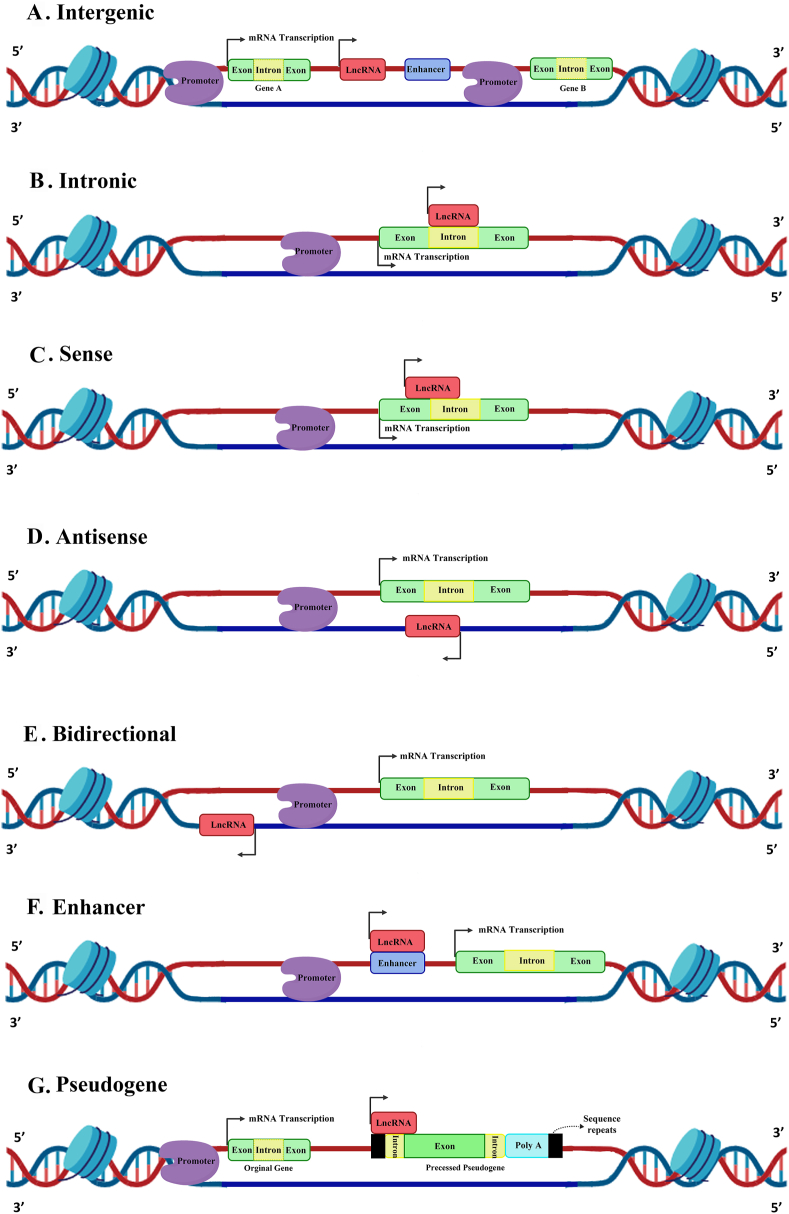
lncRNA classification according to genomic location. **(A)** Intergenic lncRNAs are located between two protein-coding genes. **(B)** Intronic lncRNAs are derived from introns of protein-coding genes. **(C)** Sense lncRNAs are transcribed in the same direction and strand from exons and introns of protein-coding genes. **(D)** Antisense lncRNAs are transcribed in the opposite direction. **(E)** Bidirectional lncRNAs are located near the promoter associated with protein-coding genes but are transcribed from the opposite strand. **(F)** Enhancer lncRNAs. **(G)** Pseudogene lncRNAs are a group of lncRNAs derived from pseudogenes.

Cell-type- and stage-specific stimuli regulate the cell-specific biogenesis of lncRNAs.^108^ RNA polymerase II (Pol II) is responsible for transcribing the majority of known lncRNAs from various DNA elements, such as enhancers, promoters, intergenic regions, and exonic regions.^109^^,^^110^ Therefore, the structure of lncRNAs is similar to mRNA, and they often have poly-A tails and caps. Different lncRNA isoforms can be generated from the same locus by processes such as alternative cleavage, alternative polyadenylation, and alternative splicing.^109^^,^^111^, ^112^, ^113^ Based on functional research, lncRNAs have four distinct mechanisms by which they regulate the expression of genes: signal, guide, scaffold, and decoy (Fig. 1).^109^^,^^114^^,^^115^ In response to stimuli, signal lncRNAs are expressed at specific locations and times within the cell. These lncRNAs regulate gene expression by acting as signals for various molecules, including transcription factors, throughout the process. On the other hand, guide lncRNAs regulate target gene expression by interacting with ribonucleoprotein complexes and chromatin-modifying enzymes, directing them to specific genomic regions. Also, some lncRNAs drive histone modifications and gene expression by serving as scaffolds for forming ribonucleoprotein complexes. At last, decoy lncRNAs facilitate activating or repressing their target genes by acting as a sponge for miRNAs, transcription factors, or RNA-binding proteins. Distinct expression of lncRNAs occurs in malignant processes like cancer and normal biological processes, including cell differentiation, growth, and imprinting.^116^ lncRNAs play an important role in the progression of various cancers by regulating the biogenesis of CSCs.^117^ For instance, lncTCF7 activates the Wnt signaling pathway to enhance the self-renewal capacity of liver CSCs.^118^ lncRNAs are categorized based on the locus of their biogenesis: intergenic lncRNAs, intronic lncRNAs, antisense lncRNAs, sense lncRNAs, bidirectional lncRNAs, and enhancer lncRNAs (eRNAs)^119^; in addition, transcribed pseudogenes constitute a unique class of lncRNAs (Fig. 2).

## Pseudogene-derived lncRNAs and their biological function

There are three types of pseudogenes based on their distinct biogenesis processes: processed pseudogenes (mRNA reverse transcription and genomic integration), unprocessed pseudogenes resulting from incomplete gene duplications, and unitary pseudogenes (gene inactivation caused by various mutations).^120^ Despite their abundance in the human genome, only a small proportion of all three types of pseudogenes are transcribed.^32^ Recent research shows that several pseudogenes with established biological functions are transcribed into lncRNAs.^32^ Of note, the transcripts of pseudogenes can regulate their target gene expression in the post-transcriptional phase by acting as antisense RNAs, endogenous small-interference RNA (endo-siRNA or siRNA), and ceRNAs, and interacting with RNA-binding proteins.^121^, ^122^, ^123^, ^124^ When RNAs compete with one another for the same miRNA, they can influence each other's expression via sharing miRNA response elements; this is the reason why they are also called ceRNAs.^41^ According to this theory, pseudogene-derived lncRNAs can regulate gene expression by acting as sponges for miRNA.^125^^,^^126^ For instance, it has been proven that the lncRNA double homeobox A pseudogene 8 (DUXAP8) regulates the Ras-related protein 14 (*RAB14*) oncogene by sponging up miR-577.^127^ In liver cancer, the pseudogene OCT4-pg4 transcript has been shown to act as a ceRNA, competing with miR-145 to regulate *OCT4* expression.^128^ Pseudogene-derived lncRNAs are functionally necessary for a variety of biological processes, including cell cycle, proliferation, migration, and apoptosis, as well as the progression and stemness of cancer.^129^, ^130^, ^131^ Remarkably, the research has demonstrated that some pseudogene-derived lncRNAs regulate their protein-coding genes in either sense or antisense forms. This unique mechanism has been observed in various tumor types.^132^^,^^133^ An interesting example in this regard is the positive and negative regulation of the tumor suppressor gene *PTEN* (phosphatase and tensin homolog) by PTENP1 (a sense transcript of pseudogenes) and PTENP1-AS1 (an antisense transcript of pseudogenes), respectively.^134^

## Dysregulated pseudogene-derived lncRNAs and the development of CSCs in different human cancers

**Figure 3:**
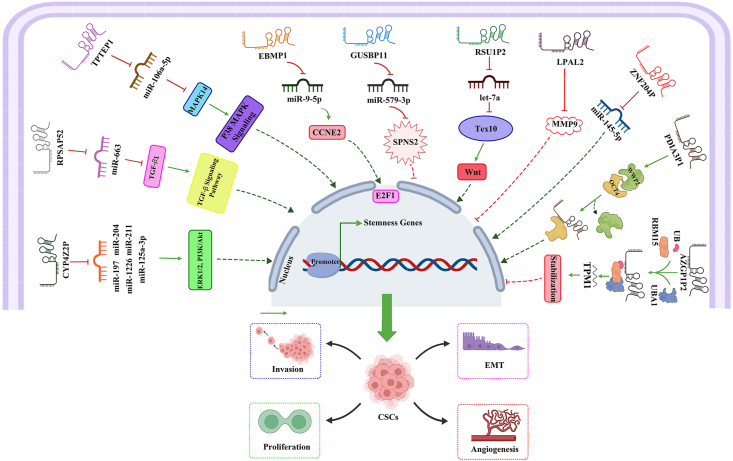
Schematic representation of the regulatory function of pseudogene-derived lncRNAs involved in the development of cancer stem cells (CSCs) in various cancers. Research indicates that pseudogene-derived lncRNAs regulate CSCs by functioning as ceRNAs for miRNAs and RNA-binding proteins (RBPs), altering downstream signaling pathways, and regulating the stemness of cancers as either oncogenes or tumor suppressors. CYP4Z2P, cytochrome P450 family 4 subfamily Z member 2, pseudogene; RPSAP52, ribosomal protein SA pseudogene 52; TPTEP1, transmembrane phosphatase with tensin homology pseudogene 1; EMBP1, embigin pseudogene 1; GUSBP11, glucuronidase beta pseudogene 11; RSU1P2, Ras suppressor protein 1 pseudogene 2; LPAL2, lipoprotein(A)-like 2; ZNF204P, zinc finger protein 204, pseudogene; PDIA3P1, protein disulfide isomerase family A member 3 pseudogene 1; AZGP1P2, alpha-2-glycoprotein 1, zinc-binding (AZGP1) pseudogene 2; RBM15, RNA binding motif protein 15; UBA1, ubiquitin-like modifier activating enzyme 1; PI3K, phosphoinositide 3-kinase; AKT, protein kinase B; ERK1/2, extracellular signal-regulated kinase 1/2; TGF-β, transforming growth factor-beta; MAPK, mitogen-activated protein kinase; CCNE2, cyclin E2; SPNS2, sphingolipid transporter 2; Tex 10, testis-expressed protein 10; MMP9, matrix metallopeptidase 9; OCT4, octamer-binding transcription factor 4; TPM1, tropomyosin 1.

Pseudogene-derived lncRNAs normally implement cell programs dynamically in response to different physiological conditions by regulating the transcription of parental and unrelated genes. They also act as key regulators of cancer cell stemness in different human cancers by either activating or inhibiting the development of CSCs via stimulating or impeding various regulatory signaling pathways related to CSCs. Their main functional role in this regard is ceRNA activity. In this section, we will describe the dysfunction of different pseudogene-derived lncRNAs linked to the development of CSC in various human tumors by influencing one or more regulatory signaling pathways. Table 1 and Figure 3 provide an overview of the contribution of diverse pseudogene-derived lncRNAs with their abnormal mechanisms in the development of CSC in different types of cancers.

**Table 1:** Pseudogene-derived lncRNAs and their regulatory functions in the development of CSCs.

Pseudogene-derived lncRNA	Cancer type	Function	Mechanism of action	Target	Pathway	Reference
CYP4Z2P	Breast	Promotes the stemness of breast cancer cells	ceRNA	miR-211, miR-125a-3p, miR-197, miR-1226, miR-204	PI3K/Akt, ERK1/2	105
RPSAP52	Glioblastoma	Increases cancer cell stemness	ceRNA	miR-663^a^	TGF-β	145
TPTEP1	Glioma	Inhibits glioma cell stemness	ceRNA	miR-106a-5p	P38 MAPK	149
EMBP1	RCC	Increases the expression of stemness markers	ceRNA	miR-9-5p	–	159
GUSBP11	TNBC	Inhibits the stemness of TNBC cell lines	ceRNA	miR-579-3p	–	164
RSU1P2	Liver	Promotes CSC-like properties	ceRNA	let-7a	Wnt/β-catenin	169
LPAL2	HCC	Represses the stemness of HCC	Not defined	MMP9	–	171
ZNF204P	HCC	Promotes the stemness properties of HCC	ceRNA	miRNA-145-5p	–	172
PDIA3P1	ESCC	Promotes the stemness properties of ESCC	Interaction with RBPs	OCT4	–	176
AZGP1P2	Prostate	Decreases the markers of prostate CSCs	Interaction with RBPs	UBA1, RBM15	ERK1/2	104

Note: ^a^Predicted by bioinformatic analysis. ceRNA, competing endogenous RNA; RCC, renal cell carcinoma; TNBC, triple negative breast cancer; HCC, hepatocellular carcinoma; ESCC, esophageal squamous cell carcinoma; CSC, cancer stem cell; RBP, RNA-binding protein; CYP4Z2P, cytochrome P450 family 4 subfamily Z member 2, pseudogene; RPSAP52, ribosomal protein SA pseudogene 52; TPTEP1, transmembrane phosphatase with tensin homology pseudogene 1; EMBP1, embigin pseudogene 1; GUSBP11, glucuronidase beta pseudogene 11; RSU1P2, Ras suppressor protein 1 pseudogene 2; LPAL2, lipoprotein(A)-like 2; ZNF204P, zinc finger protein 204, pseudogene; PDIA3P1, protein disulfide isomerase family A member 3 pseudogene 1; AZGP1P2, alpha-2-glycoprotein 1, zinc-binding (AZGP1) pseudogene 2; MMP9, matrix metallopeptidase 9; OCT4, octamer-binding transcription factor 4; RBM15, RNA binding motif protein 15; UBA1, ubiquitin-like modifier activating enzyme 1; PI3K, phosphoinositide 3-kinase; AKT, protein kinase B; ERK1/2, extracellular signal-regulated kinase 1/2; TGF-β, transforming growth factor-beta; MAPK, mitogen-activated protein kinase.

## CYP4Z2P

In 2004, Rieger et al discovered cytochrome CYP4Z2P, a pseudogene of cytochrome P450 family 4 subfamily Z member 1 (*CYP4Z1*) gene, and showed that there was an up-regulation of both CYP4Z2P and CYP4Z1 in breast cancer.^135^ CYP4Z1 and CYP4Z2P compete with a number of tumor-suppressive miRNAs, including miR-211, miR-125a-3p, miR-197, miR-1226, and miR-204, to create a ceRNA network known as ceRNET_CC.^136^, ^137^, ^138^, ^139^, ^140^ ceRNET_CC suppresses apoptosis and induces angiogenesis and tamoxifen resistance.^141^, ^142^, ^143^ Zheng et al reported that breast cancer tissues had higher expression levels of transcriptional factor SIX homeobox 2 (six 2), CYP4Z1, and CYP4Z2P.^105^ Combining *in vitro* and *in vivo* research with signaling pathway analysis and RNA sequencing has revealed that ceRNET_CC can increase the stemness of breast cancer cells by activating the PI3K/Akt and ERK1/2 signaling pathways. Furthermore, it is demonstrated that six2 can increase the expression of CYP4Z1 and CYP4Z2P and also activate ceRNET_CC by binding to their promoters, which in turn promotes the stemness of breast cancer cells. The regulatory effect of six2 on the breast CSCs can result in ADRIAMYCIN resistance. Therefore, the expression profiles of six2, CYP4Z1, and CYP4Z2P can predict chemotherapy sensitivity in patients with breast cancer.^105^ Therefore, six2/ceRNET_CC seems to be a promising potential therapeutic option to target breast CSCs.^105^

## RPSAP52

D'Angelo et al demonstrated that gonadotrophs and prolactin-secreting pituitary adenomas had significantly higher levels of ribosomal protein SA pseudogene 52 (RPSAP52), a new antisense lncRNA for the *HMGA2* gene.^144^ In this study, RPSAP52 was found to expedite the transition G1-S phase of the cell cycle, which in turn stimulated cell proliferation.^144^ Wang et al reported that glioblastoma tissues have higher expression of RPSAP52 and transforming growth factor beta 1 (TGFB1) compared with normal tissues.^145^ Additionally, high expression of RPSAP52 was negatively correlated with overall survival and positively correlated with TGFB1 expression levels. The results of the cell stemness assay indicated that silencing and overexpression of RPSAP52 led to a decreased and increased number of CD133^+^ cells, respectively. Based on bioinformatics findings, RPSAP52 can positively regulate TGF-β1 by acting as a sponge for several miRNAs, including miR-663.^145^ Finally, RPSAP52 may be a potential therapeutic target for glioblastoma given its positive regulatory function on TGF-β1.

## TPTEP1

Transmembrane phosphatase with tensin homology pseudogene 1 (TPTEP1) has a tumor suppressor role in lung and hepatocellular carcinoma and participates in several biological processes, such as cell proliferation and chemoresistance.^146^, ^147^, ^148^ In one study, through the combination of *in situ* hybridization and analyzing RNA-sequencing data available in The Cancer Genome Atlas (TCGA) database, it was demonstrated that the expression level of TPTEP1 was down-regulated in high-grade glioma tissues in comparison to low-grade tissues^149^; low *TPTEP1* expression showed a significant negative correlation with tumor clinicopathological features such as grade and recurrence. Both *in vitro* and *in vivo* tumor xenograft studies confirmed that TPTEP1 knockdown enhanced glioma cell stemness and radioresistance-associated gene expression, such as *OCT4*, aldehyde dehydrogenase 1 (*ALDH1*), and *γ-H2AX* (the γ phosphorylated form of the histone H2AX). In addition, the authors proposed that the effect of TPTEP1 on cell stemness and radioresistance could be caused by the ceRNA network, including TPTEP1-miR-106a-5p-MAPK14.^149^ Through the sponging of miR-106a-5p, TPTEP1 can up-regulate MAPK14 expression and activate the P38 MAPK signaling pathway. miR-106a-5p exhibits oncogenic properties in some malignancies, including colorectal and prostate cancer, by promoting radioresistance and cell stemness.^150^^,^^151^ As evidenced by the results of several studies, activation of the P38 MAPK signaling pathway has been linked to decreased stemness and radioresistance in the majority of glioblastoma patients.^152^^,^^153^ Altogether, TPTEP1 acted as a tumor suppressor by interacting with miR-106a-5p to create a reciprocal regulatory network that promoted the activation of P38 MAPK signaling in gliomas^149^; so TPTEP1 can be targeted to treat glioma and has the potential to become both a diagnostic and prognostic factor.

## EMBP1

According to bioinformatics analysis, the lncRNA embigin pseudogene 1 (EMBP1) acts as a ceRNA in renal cell cancer via the EMBP1/miR-9-5p/cyclin E2 (CCNE2) axis.^154^ The miRNA miR-9-5p can both suppress and promote tumors and is dysregulated in several cancers.^155^, ^156^, ^157^, ^158^ In renal cell cancer cell lines and tissues, EMBP1 and miR-9-5p are significantly increased and decreased, respectively, and there is a correlation between their expression and tumor grade and stage.^159^ Functional studies revealed that both EMBP1 knockdown and miR-9-5p overexpression resulted in similar results: decreased proliferation, colony formation, migration, and invasion, and increased apoptosis. Furthermore, by regulating the expression of mesenchymal and epithelial markers, including vimentin, claudin, and E-cadherin, the EMBP1/miR-9-5p axis plays a critical role in epithelial-to-mesenchymal transition. In addition to being essential for the metastasis of human cancers, epithelial-to-mesenchymal transition is strongly associated with the activity of CSCs.^160^^,^^161^ The overexpression of miR-9-5p resulted in a decreased stemness of renal cell cancer cells by suppressing the expression of the stemness markers KLF4 and Nanog. According to quantitative reverse transcription PCR and western blotting analysis, the EMBP1-miR-9-5p axis could modulate the expression of CCNE2 and the downstream effector E2F transcription factor 1 (E2F1).^159^ Moreover, the development of xenograft tumors was suppressed *in vivo* by either miR-9-5p overexpression or EMBP1 suppression; these effects were reversed by CCNE2 overexpression. In accordance with these findings, renal cell cancer tumorigenesis may be promoted by dysregulation of the EMBP1/miR-9-5p/CCNE2 axis.

## GUSBP11

lncRNA GUSBP11 (glucuronidase beta pseudogene 11) has different functions in tumor progression. GUSBP11 was up-regulated in nasopharyngeal carcinoma tissues and cells^162^; GUSBP11 knockdown inhibited the proliferation and metastasis of nasopharyngeal carcinoma cells via regulating the miR-1226-3p/transmembrane 9 superfamily member 4 (TM9SF4) axis.^162^ Analysis of RNA-sequencing and microarray data from TCGA and the Gene Expression Omnibus (GEO) databases showed that lung adenocarcinoma tissues had higher than normal levels of GUSBP11; these results were confirmed by quantitative reverse transcription PCR analysis.^163^ GUSBP11 also enhances the expression of sphingolipid transporter 2 (SPNS2) in triple-negative breast cancer cell lines and suppresses the malignancy of triple-negative breast cancer cells by sponging miR-579-3p. Wu et al found that GUSBP11 and SPNS2 were down-regulated in triple-negative breast cancer cell lines.^164^ Moreover, GUSBP11 overexpression increased apoptosis and reduced invasion, migration, and proliferation. Following GUSBP11 overexpression, the RNA and protein levels of NANOG, OCT4, and SOX2 were examined by quantitative reverse transcription PCR and western blotting, and the results showed down-regulation of these stemness markers. Additionally, they noticed that the overexpression of GUSBP11 repressed the epithelial-to-mesenchymal transition in the triple-negative breast cancer cell lines by increasing E-cadherin and decreasing the expression of matrix metallopeptidase 2 (MMP2), MMP7, N-cadherin, and vimentin proteins.^164^ Functional studies revealed that the binding of the YY1/p300/histone deacetylase 2 (HDAC2) complex to the GUSBP11 promoter is responsible for its reduced expression in triple-negative breast cancer cell lines. In conclusion, this ceRNA network may have therapeutic implications given the regulatory function of the GUSBP11/miR-579-3p/SPNS2 axis in processes including stemness, epithelial-to-mesenchymal transition, and cell proliferation.

## RSU1P2

Ras suppressor protein 1 pseudogene 2 (RSU1P2) is a pseudogene-derived lncRNA that has an oncogenic role in cervical cancer through its ceRNA interaction with miRNA let-7a (let-7a).^165^ This process has been found to stimulate angiogenesis, epithelial-to-mesenchymal transition, and proliferation.^165^ let-7a expression is decreased in several human cancers, such as prostate, breast, and gastric cancer, suggesting that this miRNA functions as a tumor suppressor^166^, ^167^, ^168^; this miRNA was down-regulated in liver cancer tissues and cell lines, whereas lncRNA RSU1P2 and testis-expressed protein 10 (Tex 10) were increased.^169^ RSU1P2 positively regulates Tex10 mRNA expression by targeting let-7a in liver cancer.^169^ Its knockdown verified a significant down-regulation of CSC-related gene expression, including ATP binding cassette subfamily G member 2 (*ABCG2*), *NANOG*, *ALDH1*, *OCT4*, and *SOX2*. This resulted in the inhibition of cell proliferation, invasion, cell viability, and epithelial-to-mesenchymal transition, as well as increased apoptosis. The oncogenic roles of RSU1P2 in liver cancer were observed in the *in vivo* experiment. It is confirmed that RSU1P2/let-7a/Tex10 regulates the Wnt/β-catenin pathway.^169^ The RSU1P2/let-7a/Tex10 axis is involved in the development of liver cancer and can be an interesting target due to its regulatory function in the expression of CSC-related genes, as well as in processes like apoptosis and epithelial-to-mesenchymal transition.

## LPAL2

Wang et al proposed that the level of lipoprotein(A)-like 2 (LPAL2) was elevated in orbital tissues and positively linked with the expression of intercellular adhesion molecule 1 (ICAM-1) and ICAM-4.^170^ This research has shown a correlation between thyroid eye disease and the LPAL2/miR-1287-5p/epidermal growth factor receptor (EGFR) axis. Lin et al also discovered that hepatocellular carcinoma tissues had lower levels of LPAL2 expression than normal tissues by examining microarray data and verifying the results using quantitative reverse transcription PCR.^171^ LPAL2 knockdown enhanced cell invasion, migration, and growth *in vitro* and xenograft tumor *in vivo* growth and weight. Bioinformatics analysis suggests that MMP9 is a target of LPAL2, which is in line with clinical observations showing a strong correlation between LPAL2 and MMP9 expression. Besides, a distinct group of hepatocellular carcinoma patients with high expression of LPAL2 and low expression of MMP9 showed a better survival rate. The results of the study conducted by Lin et al showed that LPAL2 knockdown blocked the effects of doxorubicin on cell death by suppressing the expression of apoptotic markers.^171^ Notably, suppression of LPAL2 enhanced the formation of spheres, increased the expression of markers for CSCs such as NANOG, SOX2, and lin-28 homolog A (LIN28A), and increased the distribution of CD133. These findings highlight the critical function of LPAL2 in regulating CSCs. Considering the inhibitory role of LPAL2 in the regulation of hepatocellular carcinoma stemness, this pseudogene-derived lncRNA can form the basis of a therapeutic strategy to target the CSC population.^171^

## ZNF204P

Bioinformatic analysis revealed that, compared with normal tissue, ZNF204P (zinc finger protein 204, pseudogene) is expressed at a higher level in hepatocellular carcinoma samples, conferring a poor prognosis as well as enhanced stem cell preservation and proliferation.^172^ Also, Hwang et al have demonstrated that ZNF204P knockdown inhibits cell survival, migration, and invasion, and decreases colony number. They show that as a decoy for tumor-suppressive miRNA-145-5p, ZNF204P interferes with the expression of OCT4 and SOX2, two regulators of pluripotency and self-renewal, and thereby plays an oncogenic stemness-associated role in hepatocellular carcinoma.^172^

## PDIA3P1

The up-regulation of protein disulfide isomerase family A member 3 pseudogene 1 (PDIA3P1) is reported in a variety of malignancies, including glioma, liver cancer, lung cancer, and esophageal squamous cell carcinoma.^173^, ^174^, ^175^, ^176^ Huang et al have discovered that PDIA3P1 expression is associated with the malignant characteristics of esophageal squamous cell carcinoma cells^176^; this study has shown that PDIA3P1 knockdown results in decreased migration and invasion, increased apoptosis, decreased colony formation, and reduced proliferation.^176^ Implicating flow cytometric analysis and sphere formation assays to examine the side population (SP), CD271^+^ CD44^+^ cells, and sphere formation, they also reveal that PDIA3P1 enhances CSC features of esophageal cancer. Furthermore, they show that PDIA3P1 binds to OCT4, inhibiting its ubiquitination by WW domain-containing E3 ubiquitin protein ligase 2 (WWP2); subsequently, OCT4 binds to *PDIA3P1*'s promoter and enhances its expression. Together, PDIA3P1 and OCT4 establish a positive feedback network that regulates the CSC characteristics of esophageal cancer. In this way, PDIA3P1 may be a potential therapeutic target in treating esophageal cancer.

## AZGP1P2

Alpha-2-glycoprotein 1 zinc-binding (AZGP1) encodes zinc-alpha-2-glycoprotein, and lower expression levels of this gene contribute to a higher death rate in castration-resistant prostate cancer.^177^, ^178^, ^179^ AZGP1P2 is an unprocessed pseudogene of AZGP1. Prostate CSCs and castration-resistant prostate cancer cell lines have down-regulated levels of AZGP1P2.^104^ AZGP1P2 overexpression enhances the sensitivity of castration-resistant prostate cancer cells to docetaxel, reduces migration, increases apoptosis, and reduces prostate CSC markers, including KLF4 and SOX2, in castration-resistant prostate cancer cells. This pseudogene enhances the sensitivity to docetaxel therapy by inhibiting the ERK1/2 pathway. AZGP1P2 can bind to two RNA-binding proteins, RNA binding motif protein 15 (RBM15; an N6-methyladenosine writer) and ubiquitin-like modifier activating enzyme 1 (UBA1; an ubiquitin-activating enzyme), to form a complex that facilitates the ubiquitination and destruction of RBM15. Based on the methylated RNA immunoprecipitation assay, RBM15 regulates mRNA degradation of tropomyosin 1 (TPM1) in N6-methyladenosine methylation. TPM1 is recognized as a tumor suppressor in a range of cancer types.^180^, ^181^, ^182^ So, regarding the fact that the AZGP1P2/UBA1/RBM15-TPM1-ERK1/2 axis regulates prostate CSCs to control docetaxel treatment resistance in castration-resistant prostate cancer, it can serve as a new target for gene therapy of this cancer.^104^

### Technical approaches for investigating the role of pseudogene-derived lncRNAs in CSCs

To investigate the molecular and cellular functions of pseudogene-derived lncRNAs in CSCs, a variety of technological approaches have been used, such as high-throughput and low-throughput methods, as well as *in vitro*, *in vivo*, and *in silico* techniques.^104^^,^^105^^,^^145^^,^^149^^,^^169^^,^^172^^,^^176^ For instance, high-throughput techniques such as RNA-sequencing and microarray analysis have been utilized to identify and quantify lncRNAs. Subsequently, their expression levels have been validated using methods like quantitative reverse transcription PCR, *in situ* hybridization, or fluorescence *in situ* hybridization. Western blotting analysis is additionally employed to assess the expression levels of proteins associated with stemness. Notably, bioinformatics pipelines and data analysis techniques applied to outputs from array-based and RNA-sequencing methodologies play a pivotal role in identifying lncRNAs and interpreting associated results. Subsequently, *in vitro* gain-of-function and loss-of-function analyses, employing various targeting approaches such as siRNA, antisense oligonucleotides (ASOs), short hairpin RNAs (shRNAs), and clustered regularly interspaced short palindromic repeats (CRISPR)/CRISPR-associated protein 9 (Cas9) genome-editing technology, have been instrumental in elucidating the functional mechanisms of pseudogene-derived lncRNAs in these studies. Additionally, researchers utilize techniques such as RNA pull-down assay, RNA immunoprecipitation assay, and dual-luciferase reporter assay to examine the interactions between lncRNAs and other biomolecules, including miRNAs and proteins. Colony formation and sphere formation assays are also utilized to investigate cellular traits and properties linked to CSCs.

## Conclusion and future perspective

Cellular heterogeneity is a significant challenge in the treatment of cancer and explains resistance to therapy and relapse.^183^ Tumor recurrence, metastasis, heterogeneity, and drug resistance are features associated with a subpopulation of cancer cells known as CSCs.^9^^,^^22^^,^^184^, ^185^, ^186^ Some of the most significant signaling pathways involved in the regulation and development of CSCs include Wnt, JAK-STAT, TGF-β, ERK1/2, and PI3K/AKT/mTOR.

Pseudogenes were previously regarded as genomic fossils and non-functional DNA sequences; however, the next-generation sequencing method has shown that a large number of pseudogenes are actively transcribed. A growing body of evidence suggests that by acting as endo-siRNA, antisense RNA, and competitors for RNA-binding proteins and miRNA, pseudogenes play important roles in regulating gene expression at the transcriptional and post-transcriptional levels.^125^ Thus, these pseudogene-derived transcripts are potentially important for the pathogenesis and progression of some diseases, such as cancer.

Pseudogene-derived lncRNAs are non-coding transcripts of pseudogenes that have a length of more than 200 nucleotides. Many investigations have shown that they can affect the progression or inhibition of various cancers by acting as ceRNA.^187^ Based on the ceRNA mechanism, lncRNAs originating from pseudogenes can control the expression of both parental and non-parental genes by interacting with miRNAs through shared miRNA response elements.

Recently, functional investigations have revealed that one of the main regulators of CSCs is pseudogene-derived lncRNAs. This review provided an overview of the regulatory roles that pseudogene-derived lncRNAs play in the development of CSCs. These transcripts interact with miRNAs and mRNAs to form a ceRNA network, which in turn regulates signaling pathways and intracellular and extracellular markers associated with CSCs. Thus, this special class of lncRNAs either promotes or inhibits the stemness of different types of cancer. Future studies, leveraging advancements in *in vitro* techniques such as next-generation sequencing (*e.g.*, single-cell sequencing and RNA sequencing), alongside the increasing availability of large-scale datasets in repositories like TCGA and GEO, are anticipated to identify additional pseudogene-derived lncRNAs. Moreover, integrative multi-omics approaches are expected to unveil novel dimensions of the roles of these lncRNAs in CSC biology and development. In addition to *in vitro* studies, the functions of some of these pseudogene-derived lncRNAs have been investigated *in vivo* using xenograft models. As an example, research has shown that the overexpression of CYP4Z1 and CYP4Z2P is associated with increased tumor size and weight, whereas their knockdown leads to decreased tumor formation potency.^105^ Nevertheless, more *in vitro* and *in vivo* studies are required to understand the roles and functions of pseudogene-derived lncRNAs in regulating CSCs, as well as to determine their significance as therapeutic targets in the treatment of cancer.

## CRediT authorship contribution statement

**Seyed Taha Nourbakhsh:** Writing – review & editing, Writing – original draft. **Fatemeh Mohamadhashem:** Writing – review & editing. **Elahe Soltani Fard:** Visualization. **Faezeh Mohamadhashem:** Writing – review & editing. **Abdolreza Daraei:** Conceptualization, Writing – review & editing.

## Conflict of interests

The authors declared no conflict of interests.
